# Perianal Necrotizing Fasciitis with Retroperitoneal Extension: A Case Report from Pakistan

**DOI:** 10.7759/cureus.5052

**Published:** 2019-07-01

**Authors:** Mirza Zain Baig, Abeer Aziz, Umm E Hani Abdullah, Mohammad Salman Khalil, Sumiya Abbasi

**Affiliations:** 1 Surgical Oncology, Rudy L. Ruggles Biomedical Research Institute, Danbury, USA; 2 Surgery, Aga Khan University Hospital, Karachi, PAK; 3 Neurosurgery, Aga Khan University Hospital, Karachi, PAK; 4 Epidemiology, Liaquat College of Medicine & Dentistry, Karachi, PAK

**Keywords:** necrotizing fasciitis, retroperitoneum, perianal, fournier’s gangrene

## Abstract

Necrotizing fasciitis is a rare but potentially fatal condition. It is defined as a rapidly spreading infection of the subcutaneous soft tissue. Extension into the retroperitoneum may further complicate this deadly condition. We report a case of a 45-year-old gentleman who presented to our institute with perianal necrotizing fasciitis with extension into the retroperitoneum. He was managed with antibiotics and prompt surgical debridement. Our patient had a positive outcome which may be due to the fact that we had a high clinical suspicion, on the basis of which we opted for early operative management rather than delaying definitive treatment by obtaining imaging.

## Introduction

Necrotizing fasciitis is a rare but life-threatening condition [1, 2]. It is a rapidly spreading infection of the subcutaneous tissue which may ensue in septic shock hence giving very high mortality [3]. Median mortality rates are reported as 32.2% [3]. We report a rare case of perianal necrotizing fasciitis with retroperitoneal extension, successfully treated at our institute.

## Case presentation

A 45-year-old gentleman presented to our emergency department with a five-day history of perianal discharge, a three-day history of high-grade fever and a one-day history of acute shortness of breath. The patient revealed that he had felt something protruding around his perianal region and had previously consulted a traditional healer who had prescribed an ointment - the contents of which were not identified. The ointment had provided him no relief.

**Figure 1:**
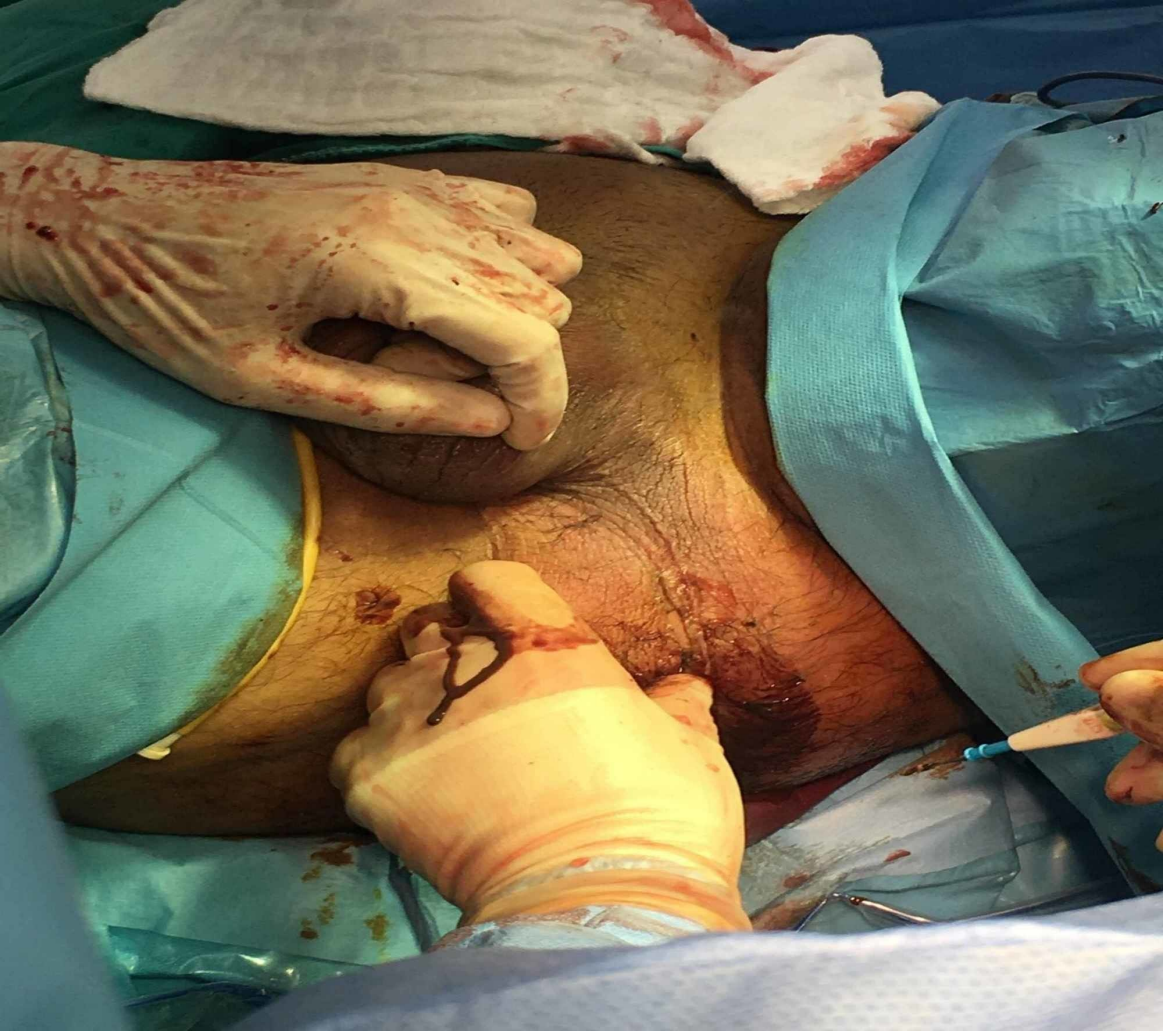
Small thumb-sized lesion being evaluated by the surgeon on call.

He had a respiratory rate of 42 breaths/minute, a pulse of 132 beats/minute, blood pressure of 111/50 mmHg and a temperature of 39.5 degrees Celsius. Chest auscultation revealed bilateral equal air entry. The abdomen was firm, distended and non-tender with audible gut sounds. He was drowsy with a Glasgow coma scale (GCS) score of 12/15. Local examination revealed foul smelling, small necrotic patch on his perianal region with purulent discharge (Figure 1). The patch was tender and swollen and there was associated erythema extending to the entire perineal region. Suspecting a case of necrotizing fasciitis, the patient was rushed to the operating room for debridement.

**Figure 2:**
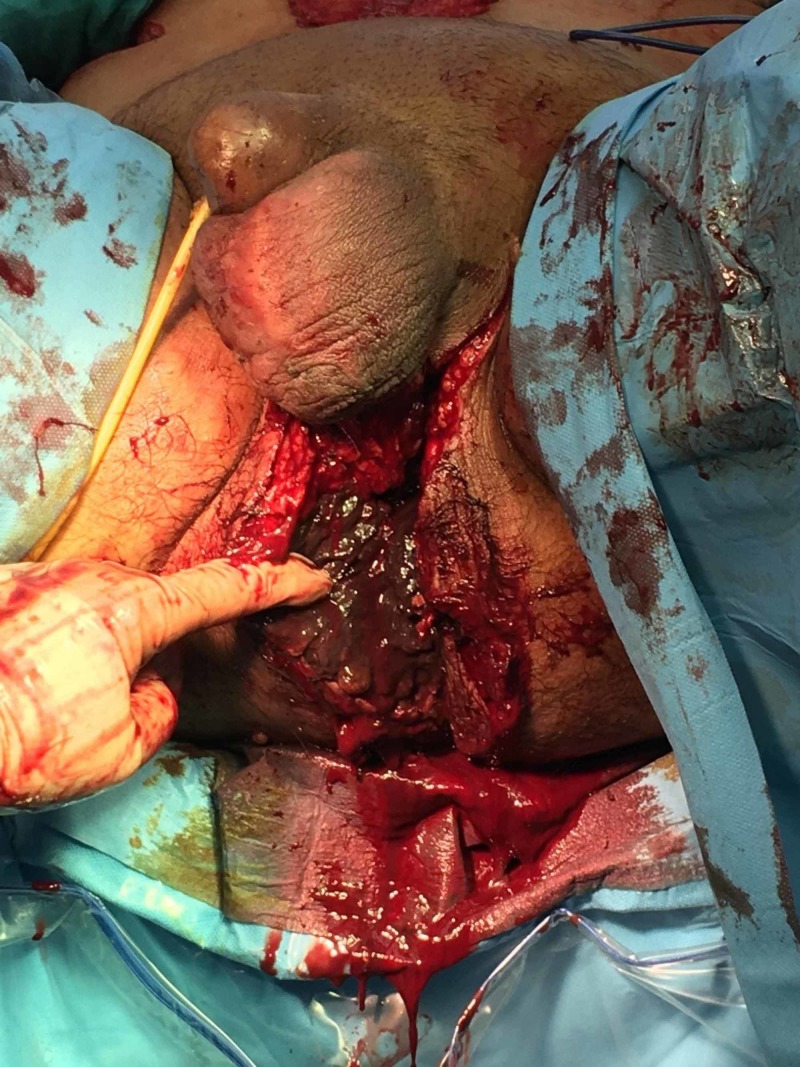
Debridement of the lesion revealing purulent necrotic exudate extending up to and beyond the ischiorectal fossae and communicating with the retropubic space.

During local debridement, necrotic foul-smelling pus was found throughout the ischiorectal fossae bilaterally and was communicating between the retropubic spaces (Figure 2). It was also involving the retroperitoneal spaces on both sides. A midline laparotomy was also performed with thorough irrigation, along with bilateral flank incisions and drainage of pus. A diverting colostomy was attempted, however, there was continuous drainage of pus from the abdominal cavity. A relook debridement procedure was performed 24 hours later to remove any further necrotic tissue, irrigate the involved areas and remove any remaining pus. The rectus sheath was closed and a transverse loop colostomy was formed. A final debridement of the perineal wound was carried out nine days later with a washout of the abdominal cavities and spaces with peroxide.

The patient was transferred to the surgical intensive care unit (SICU) where he remained intubated for the next three days. The patient was administered meropenem, vancomycin, and clindamycin during the procedure and afterward in the SICU as well to address ongoing sepsis. For pain control morphine was administered for the first two days then switched to tramadol.

After the first two days in the SICU, renal function started deteriorating and a diagnosis of acute kidney injury secondary to sepsis was made. He was started on hemodialysis and vasopressor support. The patient remained in the SICU for 15 days and after successful reduction of creatinine to near baseline values, the patient was discharged after a total hospital stay of 25 days.

## Discussion

Necrotizing fasciitis is a fulminant necrotizing disease of the soft tissue with devastating consequences. It is generally found in obese, diabetics, immunocompromised, alcoholics and patients with peripheral vascular disease, chronic renal failure, systemic lupus erythematosus, liver cirrhosis and Addison’s disease [3]. However, necrotizing fasciitis has been known to occur in young individuals also, who have none of the above risk factors [3]. This was the case with our patient. It is difficult to isolate any predisposing factors in our case. The patient's application of the ointment acquired from the traditional healer might have played a role.

Our patient’s presentation raised a high suspicion of necrotizing fasciitis. We, therefore, decided to forego any imaging so as to not delay definitive operative treatment. We believe this significantly contributed to our patient's survival.

In our patient, necrotizing fasciitis spread into the pelvis and retroperitoneum from the perianal region. Access to the internal pelvis from the perineum is difficult particularly in the well-vascularized deep recesses due to the risk of vascular injury and hemorrhage. A ventral approach via the anterior midline may be required. Retroperitoneal spread also requires abdominal exploration with colonic medial visceral rotation for exposure of the posterior abdominal wall and organs [4].

Our center previously reported two more cases of retroperitoneal necrotizing fasciitis [2]. Both patients had perianal procedures (an incision and drainage of perianal abscess and a lateral sphincterotomy for anal fissure) done at a different facility which may have served to introduce the infection. Though both were relatively young, 42- and 32-year-old respectively like our patient above, they had underlying risk factors of diabetes mellitus and obesity present. Their clinical profiles are present in Table 1.

**Table 1:** Summary of the previous two cases of retroperitoneal necrotizing fasciitis reported from our center by Alvi and Shamsi.

Patient	Comorbid	Inciting event	Examination	Lab findings	Computerized tomography	Treatment	Outcome
42, Male	Diabetes mellitus type 2	Incision and drainage of perianal abscess	Toxic looking, tachypnea, tachycardia, abdominal distension, perineal and scrotal swelling. 2 x 2 cm wound in the left lateral position	Pancytopenia, azotemia, hyponatremia	Extensive soft tissue edema, fluid pockets, and free gas in retroperitoneum	Resuscitation and administration of broad-spectrum antibiotics was done. Surgical exploration and debridement done. A transverse loop colostomy was created. Relook laparotomy after 48 hours was done. He developed bleeding from colostomy at day 40. Embolization of right colic artery done.	The patient was discharged upon recovery at day 60.
32, Male	Obesity	Lateral sphincterotomy for anal fissure	Tachypnea, tachycardia, hypotension, cold and clammy extremities, abdominal distension with right-sided tenderness, perineal and scrotal swelling. Tenderness around sphincterotomy wound.	Leukopenia, metabolic acidosis, elevated troponin	Diffuse inflammatory changes within the peritoneal cavity and focal dilation of jejunal loops	Patient was moved to the cardiac care unit for resuscitation and broad-spectrum antibiotics. Surgical exploration and debridement was done. Multiple open drains in the pelvis and retroperitoneum were created. Relook laparotomy after 24 hours was done.	After a period of stay on ventilator and pneumonia, he achieved recovery and discharged on day 25.

## Conclusions

We report a case of necrotizing fasciitis with retroperitoneal extension that was treated with success at our institute. We believe the quick diagnosis and definitive treatment in surgical debridement was the main reason why our patient had a positive outcome. Necrotizing fasciitis should be a clinical diagnosis, with dependence on imaging only in ambiguous cases as delay in operative treatment is known to increase mortality.
